# Effects of Vitamin E on the Gut Microbiome in Ageing and Its Relationship with Age-Related Diseases: A Review of the Current Literature

**DOI:** 10.3390/ijms241914667

**Published:** 2023-09-28

**Authors:** Dashine Gothandapani, Suzana Makpol

**Affiliations:** Department of Biochemistry, Faculty of Medicine, Level 17 Preclinical Building, Universiti Kebangsaan Malaysia, Jalan Yaacob Latif, Bandar Tun Razak, Cheras, Kuala Lumpur 56000, Malaysia; dashine.gothandapani@gmail.com

**Keywords:** vitamin E, gut microbiome, dysbiosis, ageing, age-related disease

## Abstract

Ageing is inevitable in all living organisms and is associated with physical deterioration, disease and eventually death. Dysbiosis, which is the alteration of the gut microbiome, occurs in individuals during ageing, and plenty of studies support that gut dysbiosis is responsible for the progression of different types of age-related diseases. The economic burden of age-linked health issues increases as ageing populations increase. Hence, an improvement in disease prevention or therapeutic approaches is urgently required. In recent years, vitamin E has garnered significant attention as a promising therapeutic approach for delaying the ageing process and potentially impeding the development of age-related disease. Nevertheless, more research is still required to understand how vitamin E affects the gut microbiome and how it relates to age-related diseases. Therefore, we gathered and summarized recent papers in this review that addressed the impact of the gut microbiome on age-related disease, the effect of vitamin E on age-related disease along with the role of vitamin E on the gut microbiome and the relationship with age-related diseases which are caused by ageing. Based on the studies reported, different bacteria brought on various age-related diseases with either increased or decreased relative abundances. Some studies have also reported the positive effects of vitamin E on the gut microbiome as beneficial bacteria and metabolites increase with vitamin E supplementation. This demonstrates how vitamin E is vital as it affects the gut microbiome positively to delay ageing and the progression of age-related diseases. The findings discussed in this review will provide a simplified yet deeper understanding for researchers studying ageing, the gut microbiome and age-related diseases, allowing them to develop new preclinical and clinical studies.

## 1. Introduction

Ageing is an inevitable process in all living organisms, accompanied by the progressive reduction of physiological function and physical deterioration and a greater likelihood of disease and mortality [1,2]. At the biological level, the accumulation of a broad spectrum of damage at the systemic, molecular and cellular levels over time leads to ageing resulting in a steady reduction in mental and physical ability, a rise in illness risk and ultimately, mortality [1]. Previous studies have identified 10 hallmarks or distinctive ageing characteristics in these three levels. At the molecular level, the hallmarks are epigenetic alterations, genomic instability, telomere dysfunction, loss of proteostasis, mitochondrial dysfunction and compromise of autophagy. At the cellular level, the discovered hallmarks are the exhaustion of stem cells, intercellular communication and cellular senescence, whereas at the systemic level, the hallmark is deregulated nutrient sensing [1,3,4,5,6].

A normal ageing process is linked to an imbalance in the gut microbiota homeostasis. The disruption of the commensal balance between the host and gut microbiome is known as dysbiosis. Dysbiosis leads to a disruption in the gut microbiota function, in relation to the host. The host’s gut microbiota is engaged in numerous biological and physiological functions, which include gut motility regulation, prevention of foreign pathogen colonization in the gut, neurotransmitter secretion, strengthening of gut integrity or development of intestinal mucosa and host immunity regulation [7,8]. Moreover, the gut microbiota is also involved in nutrient extraction from food, metabolism and the biosynthesis of bioactive molecules, including vitamins, amino acids and lipids [9]. Hence, the host’s health will also be affected by dysbiosis [7,10]. The abundance of gut microorganisms varies depending on the host’s physical condition. During ageing, the gut microbiota composition experiences some alterations, leading to a decrease in gut microbiota diversity. This then results in a decline in beneficial bacteria levels, for instance, *Bifidobacterium*, and a rise in pathological bacteria levels, such as *Proteobacterium*. A study in China discovered that old individuals were enriched with some bacteria, including *Escherichia coli*, *Parabacteroides distasonis* and *Ruminococcus gnavus* compared to young individuals. The high abundance of these bacteria was linked to the increase in chronic inflammation due to the elevation in pro-inflammatory cytokine levels, thereby leading to the reduction in lymphocyte levels, natural killer cell levels and phagocytic activities [11]. Another study which was conducted in Korea stated that young individuals had higher levels of *Lactobacillus* while old individuals were enriched with *Escherichia* [12]. Hence, this evidence showed that ageing does influence the gut microbiota composition. Gut dysbiosis, which is caused by the alterations in the gut microbiome, leads to the progression of many age-related diseases in the elderly. Some examples of age-related diseases are irritable bowel syndrome (IBS), diabetes mellitus, sarcopenia, Parkinson’s disease (PD), inflammatory bowel disease (ulcerative colitis and Crohn’s disease), cancers, Alzheimer’s disease (AD) and atherosclerosis [13,14].

Besides ageing, there are also other factors affecting the gut microbiome which are diet, medication, race, genetics, socioeconomic status, geographical location, body mass index, smoking, infection and illnesses [7,10]. Elie Metchnikoff was the first person to emphasize the gut microbiota’s importance to human health and ageing when he hypothesized that the leaking of “putrefactive bacterial autotoxins” from the colon induced senility. To combat these autotoxins, he recommended consuming fermented milk and leading a “simple” lifestyle [15].

It has been shown that ageing can be slowed down by many interventions, and by delaying ageing, the progression of age-related diseases can also be slowed down or prevented [16]. The economic burden of age-linked health issues increases as ageing populations increase, hence an improvement in disease prevention or therapeutic approaches is urgently required [6]. Substantial research has been performed to determine the possible interventions to delay ageing and age-related diseases, including probiotics, prebiotics, fecal microbiota transplant (FMT) and vitamins [7]. According to the International Scientific Association for Probiotics and Prebiotics (ISAPP), probiotics are live microorganisms that provide beneficial effects to the health of the host when administered in sufficient amounts while prebiotics are utilized by microorganisms selectively, resulting in a health benefit [17]. Probiotic and prebiotic consumption is able to restore the imbalanced composition of the gut microbiota. However, there are some cases reported on the occurrence of sepsis and bacteremia induced by *Lactobacillus rhamnosus GG*, *Lactobacillus casei*, *Bacillus clausii* and *Bacillus subtilis* [18]. Moreover, FMT is an intervention that involves transplanting the functional microbiota from donor feces into a patient’s gastrointestinal tract to change and restructure their gut microbiome [19]. However, there were two cases of transmission of drug-resistance extended-spectrum beta-lactamase (ESBL) *E. coli* which causes bacteremia through FMT and one of the patients died from severe sepsis. Before the transplantation, the donor was screened according to the Food and Drug Administration (FDA) and was considered healthy [20]. Hence, this shows that although FMT as well as probiotics and prebiotics had benefited many people with age-related disease before, there are also chances of FMT not being successful and can lead to severe conditions, including mortality.

In recent years, vitamin E has developed many researchers’ interests in utilizing vitamin E as a potential treatment strategy in delaying ageing and the progression of age-related diseases. According to some studies, the consumption of vitamin E causes a change in the gut microbiota composition. However, there have not been much research conducted; therefore, it is still unclear how vitamin E affects the gut microbiome and how it relates to age-related diseases. Nevertheless, extensive research has been conducted on the effect of vitamin E on age-related diseases and the impact of the gut microbiome on age-related diseases. Hence, the goal of this review is to gather information regarding the influence of the gut microbiome on age-related diseases, the effect of vitamin E on the gut microbiome and the modulation of vitamin E in age-related diseases. The primary goal of this review is to illustrate the connection between vitamin E, the gut microbiome and age-related diseases caused by ageing.

## 2. Role of the Gut Microbiota on Ageing and Age-Related Disease

The term “microbiota” describes the variety of live microorganisms that exist in a specific environment, such as bacteria, fungi, viruses and other microbes. For instance, the diverse variety of microorganisms inhabiting the gastrointestinal system is termed as the “gut microbiota”. The total genetic makeup of all microorganisms that reside in the gastrointestinal system is termed as the “gut microbiome”. It encompasses not only the microbial community but also their collective genomes, structural components, metabolites and the environmental conditions that influence their activities and interactions. The human gastrointestinal tract houses a substantial microbial community consisting of approximately 100 trillion microorganisms [9,21]. The main constituents of the gut microbiome are anaerobic bacteria, which comprise thousands of species and millions of genes. The most abundant phylum found in the gut are *Firmicutes* and *Bacteroidetes* while *Actinobacteria*, *Proteobacteria*, *Verrucomicrobia* and *Fusobacteria* appear to be in small amounts. A healthy microbiota community typically exhibits characteristics such as high taxonomic diversity, a rich collection of microbial genes and a stable core microbiota [7,9]. The gut microbiota populations can be affected by numerous factors including ageing, diet, medication, race, genetics, socioeconomic status, geographical location, body mass index, smoking, infection and illnesses [7,10]. These factors contribute to dysbiosis due to the decrease in diversity and abundance of gut microbiota.

**Figure 1:**
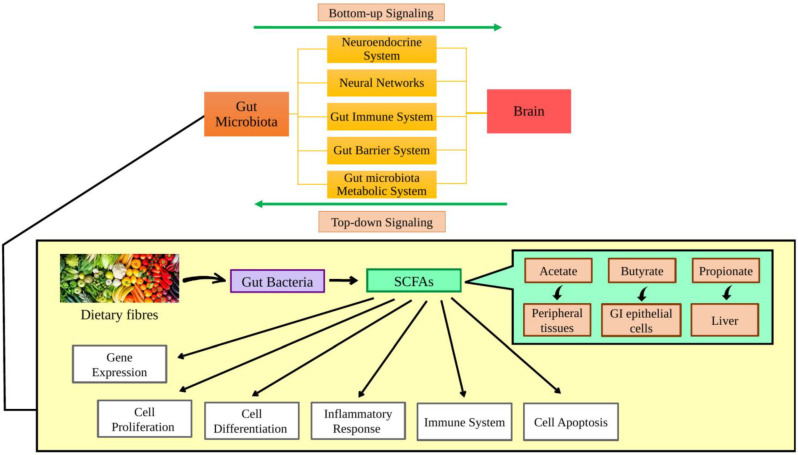
Communication pathways of BGM axis and role of SCFA in gut.

Recent studies have discovered a biochemical link between the brain and the gut bacteria, known as the “brain–gut microbiota axis” or BGM axis. The mutualistic and bidirectional connection works by “bottom-up signaling” (from digestive system to brain) or “top-down signaling” (from brain to digestive system). The gut microbiota may influence the brain through five possible communication pathways, which are the neuroendocrine system, neural networks, gut immune system, gut barrier system pathways and gut microbiota metabolic system [7,22,23]. The synthesis of beneficial metabolites in the gut microbiome, such as short-chain fatty acids (SCFAs), will also be affected negatively due to dysbiosis. Non-digestible carbohydrates, including dietary fibers, are metabolized by some bacteria in the gut to produce SCFAs. SCFAs are present dominantly in three forms which include acetate, butyrate and propionate. SCFAs are crucial in regulating multiple cellular processes, including the expressions of genes, chemotaxis, cell differentiation, cell proliferation and cell apoptosis as they are swiftly absorbed by the epithelial cells in the gastrointestinal tract (GI). Acetate is usually released into the peripheral tissues, while a large amount of propionate is absorbed by the liver, whereas butyrate is an essential SCFA for the GI’s epithelial cells as a main energy source [8,10]. Additionally, SCFAs are also engaged in regulating the immune system and inflammatory response by influencing cytokine production. As an example, the production of interleukin (IL)-18 is stimulated by SCFAs to maintain and restore epithelial integrity [8] (Figure 1).

Moreover, gut microbiota dysbiosis has also increased the permeability of the blood–brain barrier (BBB) and reduced tight junction protein expressions including claudins, tricellulin and occludin. A tightly regulated BBB is crucial to preserve a homeostatic environment which supports regular brain function. By altering tight-junction assembly and mucin production, butyrate is able to reduce bacterial translocation to the brain and at the same time improve gut barrier function by making it less permeable [8]. A study reported that SCFAs administration in mice led to the decrease in BBB permeability which is also associated with the increased expressions of tight junction proteins [24]. Another study performed on PD patients revealed that reduced abundance of *Prevotella* is correlated with increased gut permeability since low levels of *Prevotella* may imply reduced mucin production and this is linked to leaky gut as well as abnormal gut immune response which may lead to neurodegeneration [25] (Table 1).

Furthermore, dysbiosis also causes the immune system to be activated as the body produces pro-inflammatory cytokines such as IL-6, IL-8 and tumor necrosis factor-α (TNF-α) and thus promotes systemic inflammation and modulates the activation as well as the function of glial cells. When the inflammatory proteins accumulate in the brain, this then leads to a cognitive decline in ageing individuals [7]. A decrease in butyrate levels due to dysbiosis can cause dysregulation of tumor necrosis factor (TNF), C-reactive protein (CRP), IL-1β, IL-6, IL-10, chemokine ligand 5 (CCL5) and IL-2 in PD patients due to the decrease in the anti-inflammatory activities of butyrate [26]. A study on PD patients showed that *Enterobacteriaceae* was negatively associated with butyrate while *Lachnospiraceae* was positively associated with butyrate [26]. This indicates that high levels of butyrate are correlated with low levels of *Enterobacteriaceae*. The occurrence of inflammation in the gut results in the decrease in bacteria which produce butyrate and this leads to the blooming of *Enterobacteriaceae*. It was also reported that high levels of butyrate correlate with high levels of *Lachnospiraceae* and this is supported by the fact that *Lachnospiraceae* are SCFAs producers [27]. Other than that, a decrease in *Turicibacter sanguinis* relative abundance has been shown in PD patients and this indicated that the levels of butyrate also decrease in PD patients as *Turicibacter* is a butyrate-producing bacteria [28,29]. Besides butyrate, propionate was found to be in low levels in PD patients with a decline in *Bacteroidetes* and *Rikenellaceae* relative abundances [30]. Additionally, research on PD patients revealed that there was an increase in pro-inflammatory chemokine and cytokine gene expression in the gut. CRP, IL-1β, IL-1α, and IL-8 are found in the feces of PD patients with elevated levels [26] (Table 1).

A study on PD patients stated that the increase in abundance of *Verrucomicrobia* and *Bacteroides* correlates with increased levels of TNF-α and interferon (IFN)-γ in the plasma, indicating that the systemic sub-inflammatory status or low-grade inflammation occurring had connections with the alterations that took place in the gut microbiota. Furthermore, *Akkermansia* was reported to possess a few beneficial properties such as anti-metabolic syndrome and anti-diabetic properties. In addition, *Akkermansia* also offers anti-inflammatory effects where they improve the barrier function of the gut mucosal layer and the tight junction between the intestinal epithelial cells [25]. As a result, the intestinal mucosa becomes less permeable [31]. Thus, the low *Akkermansia* abundance in the gut results in the translocation of endotoxins and other pro-inflammatory products produced by pathogenic bacteria [32]. Multiple studies have examined how the gut microbiome affects AD. Firstly, a study observed that the family in phylum *Proteobacteria*, specifically *Enterobacteriaceae*, was connected to the emergence and development of AD. Members of the family *Enterobacteriaceae*, particularly *Escherichia coli*, were believed to be pro-inflammatory bacteria as the results of postmortems on the brains of AD patients showed the presence of lipopolysaccharide at elevated levels (LPS), which were the main endotoxin component of *E. coli*, in the hippocampus and neocortex as well as the perinuclear region of the brain. The accumulation of LPS will then encourage the synthesis and release of pro-inflammatory cytokines which give rise to neuroinflammatory processes, leading to neurodegeneration [33]. The high levels of *Proteobacteria* were also mentioned in another study, hence supporting the association of *Proteobacteria* with AD. This study also observed a substantial reduction in the relative abundance of SCFAs producers such as *Clostridiaceae*, *Lachnospiraceae* and *Ruminococcus* of phylum *Firmicutes*. Consequently, there was a direct association between the levels of SCFA-producing bacteria with the Montreal Cognitive Assessment (MoCA) and Mini-Mental State Examination (MMSE) scores which were the tools utilized in this study to evaluate the cognitive function of individuals with AD [34]. SCFAs produced by these bacteria are swiftly absorbed by epithelial cells in the gastrointestinal tract as a source of nutrients and at the same time, SCFAs help in reducing the mucosal barrier permeability [13,33]. Furthermore, SCFAs exhibit a positive and protective effect on the BBB and contribute to the brain’s microglial cells’ morphology maturation and immunological functions [33]. Microglial cells are vital in regulating neurogenesis, maintaining homeostasis and the brain’s cognitive function [7]. Moreover, another study also observed three other bacteria that were associated with low MMSE scores in AD patients which were *Blautia*, *Fusicatenibacter* and *Dorea*. The products secreted by these bacteria promote systemic inflammatory reactions which lead to impaired BBB and eventually cause neurodegeneration. This study also observed that in AD patients, the *Bacteroides* abundances in the gut were low. *Bacteroides* are vital in the gut to maintain the integrity of the intestinal barrier as well as to prevent a leaky gut. There were also increased levels of *Escherichia* in the feces of AD patients. *Escherichia*, specifically *E. coli*, can produce amyloid fibers that can pass through the intestinal barrier. Thereafter, the deposition of amyloid protein in the central nervous system (CNS) results in the progression of cognitive impairment and then AD [35].

Moreover, the pathogenesis of T2DM is also greatly influenced by the gut microbiome. A study discovered that there were negative associations between 10 taxa which were butyrate-producing bacteria with insulin resistance and T2DM. These 10 taxa were *Marvinbryantia*, *Peptostreptococcaceae*, *Christensenellaceae*, *Christensenellaceae R7 group*, *Ruminococcaceae NK4A214 group*, *Ruminococcaceae UCG005*, *Ruminococcaceae UCG008*, *Ruminococcaceae UCG010*, *Romboutsia* and *Intestinibacter* [36]. This discovery strengthens the growing evidence that a higher abundance of SCFAs is associated with a lower chance of developing an age-linked illness. This was also seen in another study where they observed a lower abundance of *Faecalibacterium* and *Phascolarctobacterium* in T2DM patients. *Faecalibacterium* is a butyrate-producing bacteria while *Phascolarctobacterium* is an acetate and propionate producer [37].

Moreover, an animal study on IBS discovered a decline in phylum *Bacteroidetes* and an elevation in *Firmicutes* [38]. This observation was also supported with the same finding in a clinical study [39]. The decline in beneficial bacteria was also observed in IBD individuals, including *Prevotella*, *Faecalibacterium*, *Sutterella*, *Akkermansia and Bifidobacterium.* All these bacteria produce SCFAs, thus a decline in SCFA levels is connected with the occurrence of inflammation in individuals with IBS [31].

**Table 1:** Animal and human studies on the modulation of gut microbiome in different age-related diseases.

Type of Age-Related Disease	Type of Study	Aim of Study	Study Design and Sequencing Method	Main Findings	Reference
Alzheimer’s Disease	Animal	To elucidate the effects of Ganmaidazao on AD by exploring the potential mechanism and establish the brain-gut microbiota axis to analyze the connection between gut microbiota, metabolites and AD	8-week-old adult male *Sprague Dawley* rats (6 control & 6 AD model) 16S rDNA sequencing	*Proteobacteria* ↑	[32]
	Human	To characterize the gut microbiota of amnestic mild cognitive impairment (aMCI) and AD patients	32 healthy control, 32 aMCI patients and 33 AD patients 16S rRNA sequencing	*Firmicutes* ↓ (*Clostridiaceae*, *Lachnospiraceae*, *Ruminococcaceae*) *Proteobacteria* ↑ (*Gammaproteobateria*)	[33]
		To explore the possible biomarkers before the onset of dementia and the alteration in the gut microbiota before the onset of AD and in the stage of mild cognitive impairment (MCI)	30 normal control, 30 MCI patients and 30 AD patients 16S rRNA sequencing	α-diversity ↓ *Parabacteroides* ↓ *Paraprevotella* ↓ *Alistipes* ↓ *Sutterella* ↓ *Haemophilus* ↓ *Bacteroides*↓ *Butyricimonas* ↓ *Prevotella* ↓ *Alloprevotella* ↓ *Succinivibrio* ↓ *Bifidobacterium* ↑ *Lactobacillus* ↑ *Acinetobacter* ↑ *Akkermansia* ↑ *Streptococcus* ↑ *Dorea* ↑ *Blautia* ↑ LPS ↑ IL-1 ↑ TNF-α ↑ Intestinal permeability ↑	[35]
		To examine the combination of taxonomic, functional gut microbiome and clinical data to differentiate amyloid-positive AD patients and cognitively healthy elderly controls	75 amyloid-positive AD patients shotgun metagenomic sequencing	*Aliivibrio* ↓ *Propionibacterium* ↓ *Orrella* ↓ *Veillonella* ↓ *Mucinivorans* ↓ *Paenarthrobacter* ↓ *Plesiomonas* ↓ *Roseovarius* ↓ *Lactococcus* ↓ *Sulfuricella* ↓ *Moritella* ↑ *Parabacteroides* ↑ *Basfia* ↑ *Arsenophonus* ↑ *Acidothermus* ↑ *Aureimonas* ↑ *Candidatus Arthromitus* ↑ *Asaia* ↑	[34]
Parkinson’s Disease	Animal	To examine the protective effects of osteocalcin on PD and whether the underlying mechanism is due to the alteration in the gut microbiota	Male C57BL/6 J mice (12 control and 12 PD mice model) 16S rRNA sequencing	*Bacteroidetes* ↓ *Rikenellaceae* ↓ *Erysipelotrichaceae* ↓ *Firmicutes* ↑ *Lachnospiraceae* ↑ *Clostridiales* ↑ Propionate ↓	[30]
	Human	To compare the identify the associations of the gut microbiota, gut barrier permeability, SCFAs and inflammation in PD patients and controls and to understand how these factors are connected to PD clinical symptoms	56 control subjects and 55 PD patients 16S rRNA sequencing	*Prevotella* ↓ *Firmicutes* ↑ Butyrate & propionate ↓ IL-1α ↑ IL-1β ↑ IL-8 ↑ CRP ↑	[26]
		To evaluate the effects of gut microbiota alterations and cytokines on PD patients	(1) 77 control subjects and 80 PD patients (2) 120 control subjects and 120 PD patients 16S rRNA sequencing	*Prevotella* ↓ *Parabacteroides* ↑ *Verrucomicrobia ↑* *Enterococcus ↑* *Akkermansia ↑* *Veillonella ↑* *Butyricimonas ↑* *Mucispirillum ↑* *Odoribacter ↑* *Bilophila ↑* *Lactobacillus* ↑	[25]
		To investigate the possible functional consequences due to alterations in the gut microbiota of PD patients in comparison to healthy controls	248 healthy control and 206 PD patients 16S rRNA sequencing	*Roseburia intestinalis* ↓ *Turicibacter sanguinis* ↓ *Ruminococcus bromii* ↓ *Ruminococcus torques* ↓ *Akkermansia* ↑ *Lactobacillaceae* ↑ *Christensenella* ↑ *Lactobacillus* ↑	[28]
Type 2 Diabetes Mellitus	Human	To evaluate whether clinical biomarkers along with the gut microbiota composition may enhance the prediction of new cases of T2DM in coronary heart disease patients	1002 patients with coronary heart disease 16S rRNA sequencing	*Prevotellaceae* ↑ *Carnobacteriaceae* ↑ *Veillonellaceae* ↑ *Streptococcaceae* ↑ *Actinomycetaceae* ↑ *Oxalobacteraceae* ↑	[37]
		To study the connections between the composition of the gut microbiome and insulin resistance as well as T2DM in an extensive population-based setting, taking a variety of sociodemographic and lifestyle variables into account	4671 participants 16S rRNA sequencing	*Intestinibacter* ↑ *Clostridiaceae 1* ↑ *Clostridium sensu stricto 1*↑ *Peptostreptococcaceae* ↑ *Romboutsia* ↑	[36]
Irritable Bowel Syndrome	Animal	To examine the effects of five diarrhea-predominant irritable bowel syndrome (IBS-D) rat models on the BGM axis	5 7-week-old Wistar rats 16S rRNA sequencing	*Bacteroidetes ↓* *Firmicutes ↑*	[38]
	Human	To examine the colonic melatonin levels and microbiota profiles in IBS-D patients	28 healthy controls and 32 IBS-D patients 16S rRNA sequencing	*Bacteroidetes* ↓ *Firmicutes* ↑	[39]
		To investigate the changes in gut microbiome in IBS patients, followed by the efficacy, side effects and changes in gut microbiome with FMT treatment	11 men and 6 women with IBS 16S rRNA sequencing	α-diversity ↓ *Prevotella* ↓ *Faecalibacterium* ↓ *Sutterella* ↓ *Akkermansia* ↓ *Bifidobacterium* ↓	[31]

↓ = decrease ↑ = increase.

## 3. Role of Vitamin E in Ageing and Age-Related Disease

Vitamin E is a collective term for isoprenoid chromanols. This lipophilic or fat-soluble molecule consists of eight naturally occurring forms, including α-tocopherol (αT), β-tocopherol (βT), γ-tocopherol (γT), δ-tocopherol (δT) and α-tocotrienol (αTE), β-tocotrienol (βTE), γ-tocotrienol (γTE) and δ-tocotrienol (δTE) [40]. All forms of vitamin E contain a chromanol ring, but there is an obvious distinction between tocopherols and tocotrienols. Tocopherols contain a saturated 16-carbon phytyl-like side chain while tocotrienols contain an unsaturated or geranylgeranyl-derived side chain with three double bonds [40,41].

The primary natural source of vitamin E is in the oily part of nuts and oil seeds. Tocopherols are mainly found in almond and other nut oils, sunflower oil, corn oil, olive oil, soybean oil, rapeseed oil and linseed oil [42]. Crude palm oil which is also referred to as the “tocotrienol-rich-fraction” is one of the natural sources with the most profuse tocotrienols levels. The vitamin E distribution in palm oil is 70% tocotrienols and the remaining 30% is tocopherols [43]. Tocotrienols are also found abundantly in rice bran oil and present in a small amount in coconut oil, wheat germ, annatto oil, hazelnut, barley, oat and maize [42]. Several studies have reported that vitamin E has an antioxidant, anti-inflammatory, anti-ageing and anti-cancer properties. Tocotrienols have been reported to have more potent antioxidant properties compared to tocopherol [23].

A study performed on mice with T2DM showed that tocotrienol treatment after a month had significantly decreased reactive oxygen species (ROS) levels by 50% in the red blood cells in comparison with non-treated mice. Tocotrienol treatment did not exert any impact on the glucose levels of T2DM mice. Hence, this suggests that the beneficial effects of tocotrienol on the kidney, heart and liver were not brought on by the restoration of blood sugar level, and instead likely brought on by a drop in the generation of free radicals and an improvement in mitochondrial function [44]. Another study performed on T2DM patients reported that the glycemic control, oxidative stress load and inflammatory biomarkers were all drastically improved with δTE supplementation. Chronic inflammation was said to be improved through the decrease in pro-inflammatory chemokine and cytokine production and this study reported that T2DM patients who received δTE treatment showed a substantial reduction in chronic inflammation as seen by a drop in serum TNF-α, high sensitivity C-reactive protein (hs-CRP) and IL-6 levels [45].

Furthermore, the oxidative stress level also decreased significantly as the serum malondialdehyde (MDA) level was reduced [45]. MDA is formed as an end product of lipid peroxidation of polyunsaturated fatty acids in the cell [46,47]. The structural integrity of the cell membrane is altered and undergoes degradation as lipid is the primary constituent of cell membrane [46,47,48]. The antioxidant defense system in the body can be overpowered by increasing lipid peroxidation which leads to cell death or other pathological processes. High serum MDA levels serve as a marker of increased free radical generation [46]. In T2DM, the primary causes of inflammation are hyperglycemia, hyperlipidemia and adiposity which are due to the excessive production of ROS. The generation of ROS in access and the insufficient clearance of free radicals results in cellular nucleic acids, proteins and lipid damage. When the level of ROS produced surpasses the capacity of the cell’s antioxidant defense, this results in oxidative stress [44].

In addition, Tan et al. [49] studied how vitamin E affects diabetic nephropathy which is a kidney-related complication of diabetes mellitus. Chronic hyperglycemia leads to superoxide anions being produced more rapidly in the endothelial cells’ mitochondria, resulting in diabetic nephropathy. The subsequent transformation of these superoxide anions into ROS results in the increase in advanced glycation end product (AGE) levels and the activation of pathways such as the polyol pathway flux and hexosamine pathway flux which leads to an increase in inflammation. Consequently, increasing inflammation in diabetic individuals will result in diabetic nephropathy and other macrovascular as well as microvascular complications which require a strong antioxidant and anti-inflammatory compound as an intervention and these properties are found in vitamin E. This study observed that a high dosage of tocotrienol was able to diminish the progression of diabetic nephropathy. They also reported that the supplementation of tocotrienol for 12 weeks significantly improved renal function, but no effect was observed on the glucose level which supports the finding reported by Dallner et al. [44]. Thus, the studies mentioned above proved that vitamin E was able to diminish oxidative stress levels in both T2DM patients and mice.

In addition to T2DM, there were a few studies conducted to comprehend how vitamin E affects AD. A study performed compared the effect of resveratrol which is a type of polyphenol on impaired cognitive function in scopolamine-induced rats and the effect of resveratrol in conjunction with vitamin E. This study reported that a high dose of resveratrol alone was not as beneficial as the combination of vitamin E and resveratrol. The combination of vitamin E and resveratrol had significantly restored the levels of TNF-α [50]. Hence, this showed that vitamin E was beneficial in terms of reducing the pro-inflammatory cytokine and thus improving the cognitive function. Another study reported that δTE can inhibit nuclear factor-κB (NF-κB), a protein that regulates the inflammatory responses in the body. When there are high levels of pro-inflammatory cytokines, NF-κB activation will thus be increased. This study reported that δTE was the vitamin E form that exhibited an inhibitory effect on the activated NF-κB triggered by TNF-α with the greatest impact compared to the other vitamin E forms. This finding serves as evidence for the anti-inflammatory properties possessed by vitamin E. Besides the inflammatory response, oxidative stress also impacts significantly on the initiation and development of AD [51]. Oxidative stress may boost the synthesis and accumulation of amyloid-β (Aβ) in the brain which results in synaptic and neuronal dysfunction which proceeds to cognitive impairment [52]. In addition, a study examined the effect of vitamin E on Aβ aggregation and disaggregation in vitro using human Aβ1-42 peptide. They reported that αT minimized Aβ aggregation at high concentrations which was 300 µM while αTE was able to reduce Aβ aggregation and preformed fibrils were disaggregated even at low concentrations of 10 µM. In comparison to αT and αTE, besides reducing Aβ aggregation and disaggregating preformed fibrils, γTE also diminished Aβ oligomerization, which was remarkable [52]. This study demonstrated vitamin E as a promising therapeutic approach since its antioxidant properties were able to counteract the damages brought by oxidative stress. In addition, there were a few studies reporting the correlation between dopamine and the progression of AD. The substantia nigra, ventral tegmental area and hypothalamus of the brain are where dopamine were produced [53]. Dopamine neurons were produced after a sequential hydroxylation and decarboxylation of tyrosine besides being synthesized from phenylalanine directly [54]. A study reported that reduced dopamine levels in the mice’s hippocampus were associated with the deficit in the hippocampus-dependent memory along with synaptic plasticity and reward processing impairment [55]. Another study found that dopamine was able to diminish inflammatory mediators which cause oxidative stress and inflammation induced by Aβ accumulation [56]. However, there are no studies reporting on the role of vitamin E affecting dopamine to improve AD.

Furthermore, a few studies were executed to examine how vitamin E affects colitis which is closely related to IBD and IBD is known to occur due to prolonged inflammation in the digestive tract. A study was conducted on rats that administered dextran sulfate sodium (DSS) with the intention to induce colitis and the rats were then treated with vitamin E. They noticed that the levels of pro-inflammatory cytokines reduced with 30 IU/kg αT treatment, and this included TNF-α, IL-6, IL-12 and IL-18. They also reported that αT promoted recovery and attenuated DSS-induced colitis along with the severity level of inflammation, the depth of injury and the degree of damage on the crypt of the colon tissue [57]. Moreover, another study also showed that vitamin E treatment on rats with colitis brought on by DSS had decreased the level of pro-inflammatory IL-6. Hence, these studies serve as evidence for the beneficial anti-inflammatory properties possessed by vitamin E [58].

Some studies have also mentioned the relationship between dopamine and IBD. Dopamine can be generated by both enteric neurons and non-neuronal tissues or cells, including epithelial and immune cells as well as the gut bacteria in the GI. In IBD animal models, the dopamine level was found to be decreased by around 66% [59]. Another study also detected that the levels of dopamine reduced significantly in IBD patients and that an inflamed mucus was also observed which was due to the reduction in dopamine uptake as well as the reduction in the number of sympathetic fibers that interact with the intestinal wall. In healthy individuals, high levels of dopamine in the colon can stimulate D2R which is a dopamine receptor and this could boost the synthesis of anti-inflammatory cytokine IL-10, thus preventing intestinal motility and ulcer formation. This illustrated the anti-inflammatory activity of dopamine [60]. However, as in AD, there were also no studies reporting on the role of vitamin E on dopamine which can improve IBD [61].

**Figure 2:**
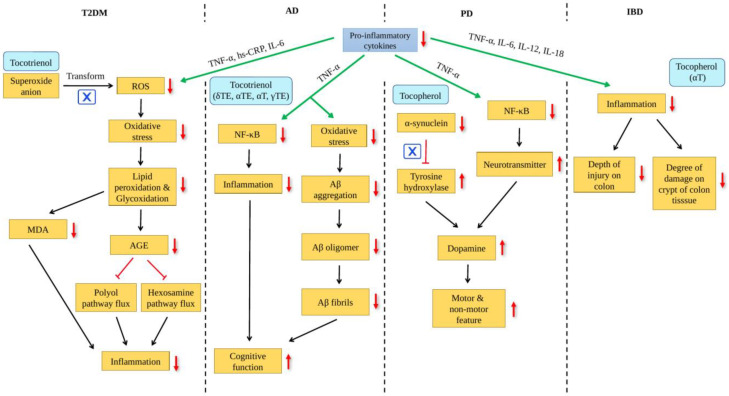
Effects of vitamin E on age-related disease. 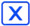 inhibition, 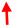: increased; 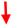: decreased.

There has also been research performed to discover vitamin E effects on PD. PD occurs when the dopaminergic neurons that exhibit both motor and non-motor features are depleted as a result of oxidative stress, neuroinflammation, α-synuclein accumulation, mitochondrial dysfunction, genetics and environmental toxins. According to a study performed on mice, it was observed that the NF-κB levels drop significantly in tocopherol-treated mice. This indicated that the levels of TNF-α also reduced as this pro-inflammatory cytokine activates an inflammatory response by activating the NF-κB pathway. In addition, the neurotransmitters levels in the brain increased, aiding in the recovery of the brain’s motor and non-motor functions. This indicates that oxidative stress levels in PD mice were reduced and neurotransmission was enhanced [62]. Furthermore, tocopherol treatment was shown to cause a decrease in mRNA expression of α-synuclein. α-Synuclein acts as an inhibitor to the tyrosine hydroxylase enzyme which is a rate-limiting enzyme and this inhibits the production of dopamine [62]. Based on a different study, they discovered that the expression of tyrosine hydroxylase increased with αTE and γTE treatments on rats injected with 6-hydroxydopamine to induce parkinsonism and the levels of dopamine neurons increased as well [63]. This shows that vitamin E was able to reduce the α-synuclein level, preventing the inhibition of the tyrosine hydroxylase enzyme and allowing the enzyme to take part in dopamine synthesis (Figure 2).

## 4. Effect of Vitamin E on Gut Microbiome and Age-Related Disease

Research on how vitamin E affects the gut microbiome has been gaining a spotlight in recent years. In recent years, utilizing natural products as a therapeutic approach has drawn greater interest from researchers. However, there are very few studies reporting on how vitamin E affects the gut microbiome and the connection with age-related diseases. A study was performed on healthy mice by studying the effect of low levels of vitamin E (LV, 0.06 mg/20 g body weights per day) and high levels of vitamin E (HV, 0.18 mg/20 g body weights per day) treatment on the gut microbiota. The ratio of *Firmicutes* to *Bacteroidetes* was the highest in the LV group, compared to the HV and control group [64]. Hence, this study managed to show that the intake of vitamin E causes alterations in the composition of the gut microbiota.

A clinical study was conducted involving healthy individuals to determine the effect of different types of vitamins including vitamin E on their gut microbiome and an in vitro study by collecting feces donated by three healthy individuals. There were no significant findings on vitamin E in the clinical study. However, from the in vitro study they found that vitamin E treatment resulted in an increased number of bacterial species which showed the influence of vitamin E on the α-diversity of the gut microbiome. Additionally, at the phylum level, vitamin E treatment increased the relative abundance of *Firmicutes*, *Actinobacteria* together with *Verrucomicrobia* and decreased the relative abundance of *Bacteroidetes,* while at the genus level, there was a boost of *Akkermansia*, *Bifidobacterium* and *Faecalibacterium* relative abundances. Moreover, vitamin E treatment led to increases in acetate, butyrate and propionate production [65]. This correlates with the increased relative abundance of SCFA-producing bacteria, including *Akkermansia* (butyrate producer) [66], *Bifidobacterium* (acetate and propionate producer) [67] and *Faecalibacterium* (butyrate producer) [68]. Additionally, they discovered that vitamin E increased transepithelial electrical resistance (TEER) which is a broadly utilized quantitative tool to evaluate the integrity of gut barrier in cellular models. A greater TEER implies a gut barrier with greater strength [65].

**Figure 3:**
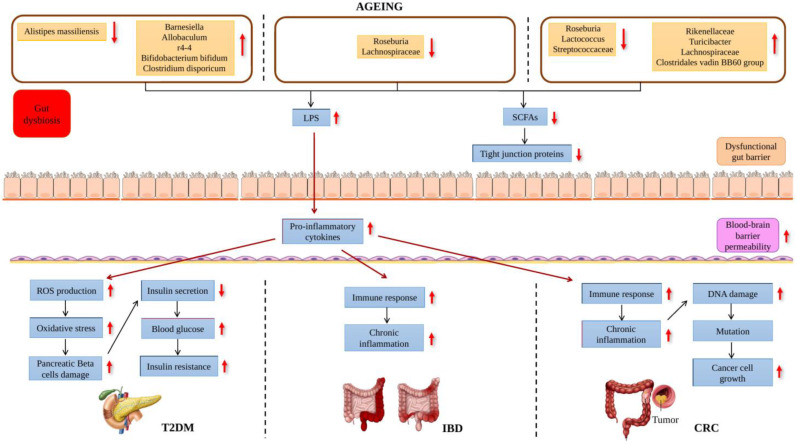
Effects of gut microbiome in ageing and its association with age-related disease. 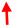: increased, 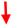: decreased.

**Figure 4:**
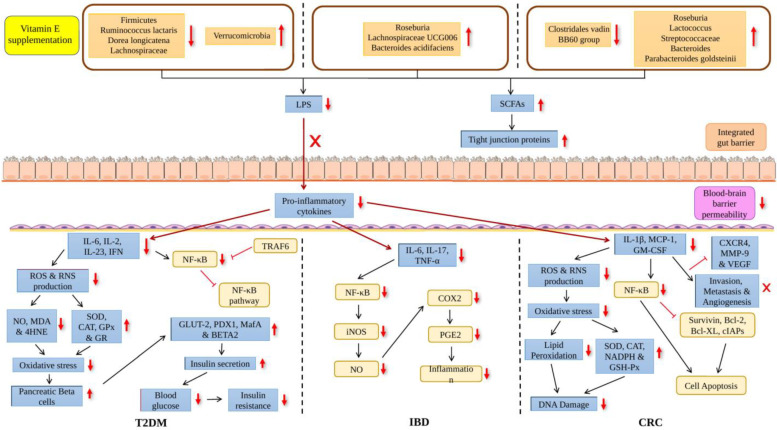
Effects of vitamin E on the gut microbiome in ageing and its association with age-related disease. **X**: inhibition, 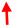: increased, 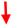: decreased.

A study on T2DM was conducted by observing three groups of mice: (1) mice fed with a low-fat diet (LFD); (2) mice fed with a high-fat diet (HFD); (3) and HFD mice supplemented with 800 mg tocotrienol/kg (AT) (Table 2). This study reported that there was a significant elevation in the *Firmicutes* to *Bacteroidetes* ratio in the HFD group in comparison to the LFD group where the increase in this ratio was shown to have a connection with HFD, obesity and T2DM. There was also a rise in *Ruminococcus lactaris* relative abundance in the HFD group. However, the relative abundance of *Clostridium disporicum*, *Bifidobacterium bifidum* and genera such as *rc4-4* and *Barnesiella* and *Allobaculum* was found to be significantly lowered (Figure 3). The group of mice supplemented with tocotrienol was found to have the highest *Verrucomicrobia* levels. Furthermore, in conjunction with the HFD group, the AT group had significantly decreased *Dorea longicatena* levels. *D. longicatena* was discovered to have a favourable correlation with the circumference of the waist and the body mass index. Hence, this shows that tocotrienol has a positive effect on people with T2DM. It was also reported that there was a significant reduction in IL-6 levels in the AT group which supports the finding of anti-inflammatory activities of tocotrienol [69] (Figure 4). Their previous study reported that the production of pro-inflammatory cytokines, including IFN, IL-2 and IL-23 was significantly elevated. However, pro-inflammatory cytokine levels were markedly reduced with tocotrienol supplementation [70]. Hyperglycemia is related to the elevated productions of ROS and reactive nitrogen species (RNS). The decrease in pro-inflammatory cytokine levels then causes a decrease in ROS and RNS production. The levels of oxidative stress biomarkers also decreased with tocotrienol supplementation, including MDA, total nitric oxide (NO) and 4-hydroxynonenal (4-HNE). Tocotrienol also restored the antioxidant activities as it increased the levels of catalase (CAT), superoxide dismutase (SOD), glutathione reductase (GR) and glutathione peroxidase (GPx). This indicated that the oxidative stress level was reduced. Glucose transporter type 2 (GLUT2) and insulin gene transcription factors such as PDX1, MafA and BETA2 also increased which leads to an increase in insulin secretion [71]. Here, we see the connection where dysbiosis occurring in the gut resulted in the elevation of proinflammatory cytokine production which eventually causes inflammation and oxidative stress in T2DM and this can be counteracted by tocotrienol supplementation. A summary of vitamin E affecting the gut microbiome and T2DM is shown in Figure 4. Inflammation and chronic oxidative stress will then lead to insulin resistance, poor insulin signaling and impaired glucose transport in the skeletal muscle of people with T2DM [70].

In T2DM patients, a study found a decline in *Faecalibacterium* and *Phascolarctobacterium* relative abundances and hence the levels of SCFAs also decreased [37]. SCFAs has the ability to protect the body against oxidative stress [72]. Thus, this illustrated that in T2DM patients, the decrease in SCFAs levels is related to the increase in oxidative stress levels. The increased in oxidative stress in T2DM patients decreased with vitamin E supplementation as mentioned by Dallner et al. [44]. Another study stated that vitamin E supplementation decreased serum TNF- α, IL-6 and hs-CRP levels in T2DM patients [45] and this also occurred due to decreased levels of SCFAs. Hence, from these studies, we can see the connection where vitamin E affects the gut microbiome by elevating *Faecalibacterium* and *Phascolarctobacterium* relative abundances which leads to the increasing levels of SCFAs. The increase in SCFA levels then causes a decrease in pro-inflammatory cytokine levels which leads to the decrease in oxidative stress levels. Hence, this leads to an improvement in T2DM pathogenesis.

Research was performed on mice to understand how vitamin E affects the gut microbiome in colitis-induced mice. In this study, mice were first treated with dextran sodium sulfate (DSS) which brought about colitis and were then supplemented with γT-rich mixed tocopherol (γTmT). γTmT had significantly decreased DSS causing the attenuation of *Roseburia* levels (Table 2). γTmT also increased certain bacteria in the *Ruminococcaceae* family as well as *Lachnospiraceae UCG006* in mice with colitis. Mice with colitis that were given αT supplementation were observed to have an increase in *Bacteroides acidifaciens* relative abundance. Hence, this suggests that vitamin E has a positive influence on the gut microbiome since it can mitigate the detrimental effect of DSS on the gut microbiome of mice. In addition, they also observed that DSS treatment caused a decline in occludin and ZO-1 levels which are the tight junction proteins measured in this study (Figure 3). There was an elevation in the level of lipopolysaccharide-binding protein (LBP) in the plasma which led to the increased intestinal barrier permeability. It is well known that LBP is strongly correlated with the leakage of the intestinal barrier which makes LBP one of the markers of gut barrier dysfunction. γTmT and αT supplementation were shown to have the ability to decrease LPS levels and increase occludin and ZO-1 levels. They also mentioned that the increase in pro-inflammatory cytokine IL-6 caused by DSS treatment had drastically decreased with γTmT and αT supplementation [58]. Other pro-inflammatory cytokines that were reported to be elevated were IL-1β, IL-12, IL-17 and TNF-α. High levels of pro-inflammatory cytokines lead to the increase in NF-κB levels and vitamin E supplementation, thus reducing both the pro-inflammatory cytokines and NF-κB levels. Eventually, the levels of iNOS, NO and COX-2 also decrease with vitamin E supplementation. The overproduction of Prostaglandin 2 (PGE2) which was due to the increase in COX-2 levels led to the increase in inflammation; however, this can be hindered by vitamin E supplementation. Moreover, tocotrienol also enhanced the non-proteasomal degradation of tumor necrosis factor receptor-associated factor 6 (TRAF6) which resulted in the early blocking of NF-κB activation. This then led to the inhibition of the NF-κB signaling pathway [73]. A summary of how vitamin E affects the gut microbiome and IBD is shown in Figure 4.

Research was performed on mice to study vitamin E’s antioxidant and anti-inflammatory activities in colorectal cancer (CRC). Azoxymethane (AOM) and DSS were administered into mice to induce tumorigenesis and were then distributed into three groups: (1) AOM/DSS-induced tumorigenesis mice (control group); (2) mice with tumorigenesis brought on by AOM/DSS supplemented with δTE; (3) and mice with tumorigenesis brought on by AOM/DSS supplemented with δTE-13′carboxychromanol (δTE-13′-COOH). δTE-13′-COOH is one of the metabolites generated after δTE is metabolized in the gut. δTE-13′-COOH supplementation reduced total polyps by 25% as well as the large-sized polyps by 55% while δTE supplementation inhibited the growth of large tumors by 34%. δTE supplementation decreased the tumor surface area by 31% while δTE-13′-COOH supplementation decreased it by 38%. In comparison to small polyps, the risk of malignancy and recurrence in humans is higher for large-sized adenomas. Thus, the ability of δTE and δTE-13′-COOH to suppress large adenomas demonstrated clinically significant cancer-prevention efficacy [74].

Treatment of AOM/DSS has shown significantly elevated levels of IL-1β, TNF, granulocyte–macrophage colony-stimulating factor (GM-CSF) and MCP-1 which indicates that pro-inflammatory cytokines were vital in the progression of CRC. It was reported that δTE-13′-COOH supplementation had significantly reduced MCP-1 and GM-CSF levels while δTE supplementation significantly reduced IL-1β levels. This serves as further evidence of vitamin E’s ability to reduce inflammation and prevent the growth of cancer. Tocotrienol had exhibited the inhibition of NF-κB activation in CRC. NF-κB was involved in the regulation of several antiapoptotic proteins such as surviving, Bcl-2, Bcl-XL and cIAPs. However, with tocotrienol, the activities of these proteins were inhibited and thus induced cell apoptosis [75]. In addition, the decrease in pro-inflammatory cytokines leads to the decrease in ROS and RNS production along with a decline in oxidative stress levels. Metabolites produced through lipid peroxidation, which was due to oxidative stress, leads to DNA damage as the metabolites can attack DNA bases. Tocotrienol was able to lower lipid peroxidation which resulted in the decline in genome instability. Tocotrienol also exhibited its antioxidant activity by inducing antioxidant enzymes, including catalase (CAT), superoxide dismutase (SOD), reduced coenzyme II (NADPH) and glutathione peroxidase (GSH-Px) [76]. Other than that, the expression of biomarkers for invasion, metastasis and angiogenic which were CXCR4, MMP-9 and VEGF, respectively, were suppressed by tocotrienol [75]. A summary of the how vitamin E affects the gut microbiome and CRC is shown in Figure 4. Moreover, the supplementation of δTE and δTE-13′-COOH resulted in a decrease in the *Firmicutes* to *Bacteroidetes* ratio which was shown to be higher in the control group. δTE-13′-COOH elevated the expressions of the genus *Roseburia* which was initially lowered by AOM/DSS treatment. *Roseburia* is a butyrate-producing bacteria which is vital in preventing inflammation of the intestine [77]. The relative abundance of *Eubacterium coprostanoligenes* was elevated while *Clostridiales vadinBB60* reduced with δTE supplementation. Both the supplementation of δTE and δTE-13′-COOH had substantially raised the relative abundance of *Streptococcaceae*, *Lactococcus* and *Parabacteroides goldsteinii CL02T12C30*. There are studies reporting that *P. goldsteinii* possesses anti-obesity and anti-virulence properties which illustrates the positive effect of this bacteria in the gut [78,79]. *Lactococcus* also exerts a positive effect in the gut, increasing the production of SCFAs [80].

Although there are limited studies reporting on the impact of vitamin E on the gut microbiome and the connection with age-related diseases, studies reporting on the influence of vitamin E on the gut microbiome and other studies reporting on the influence of vitamin E on age-related diseases have been able to show us the connections between these two areas of study. As an example, it was reported in a study that AD patients had an elevated relative abundance of *Enterobacteriaceae* which releases LPS, bringing about an increase in pro-inflammatory cytokine production and a decline in the relative abundance of SCFAs producers, including *Ruminococcus* of phylum *Firmicutes*, *Lachnospiraceae* and *Clostridiaceae* [33]. According to a different study, vitamin E substantially reduces TNF-α levels to the normal level [50]. Vitamin E also inhibited NF-κB from stimulating inflammation in the body [51]. From these studies, we are able to link that vitamin E acts upon the gut microbiome and results in the reduction in pathogenic bacteria levels such as *Enterobacteriaceae* as well as increasing the relative abundance of SCFAs producers, giving rise to the depletion of pro-inflammatory cytokine levels, and this then decelerates the progression of AD.

Furthermore, in PD patients, the rise in proinflammatory cytokine levels, including TNF-α, IFN-γ, IL-1α, IL-1β and IL-8 was associated with the decrease in *Lachnospiraceae* and the increase in *Enterobacteriaceae*, *Verrucomibia* and *Bacteroides* [25,26]. These pro-inflammatory cytokines lead to the activation of NF-κB which activates inflammatory responses [62]. As stated in another study, vitamin E significantly decreased the levels of NF-κB. From these studies, we are able to draw the connection that vitamin E affects the gut microbiome by bringing down the levels of pathogenic bacteria and increasing the beneficial bacteria levels such as *Lachnospiraceae* which leads to the increased production of butyrate. This then causes a decline in the levels of pro-inflammatory cytokines and thus NF-κB activation is prevented. The occurrence of inflammation is inhibited and thus the progression of PD is delayed.

**Table 2:** Effect of vitamin E on gut microbiome and age-related diseases.

Type of Age-Related Disease	Type of Study	Study Design	Effects of Vitamin E	Reference
Type 2 diabetes mellitus	Animal	48 male C57BL/6 J mice aged weeks (4 groups (*n* = 12 per group): low fat diet (LFD, 5% of energy from fat), high fat diet (HFD, 58% of energy from fat), HFD supplemented with 800 mg tocotrienol/kg diet (AT) and HFD supplemented with 200 mg metformin/kg diet (MET))	*Verrucomicrobia* ↑ IL-6 ↓	[69]
		58 male C57BL/6J mice of aged 5 weeks (5 group: low fat diet (LFD, 5% of energy from fat), high fat diet (HFD, 58% of energy from fat), HFD + 400 mg tocotrienol/kg diet, HFD + 1600 mg tocotrienol/kg diet and HFD + 200 mg metformin/kg diet)	IL-2 ↓ IL-23 ↓ IFN ↓	[70]
Inflammatory bowel disease	Animal	5–6-week-old male Balb/c mice (4 groups: healthy control fed with AIN93G diet (*n* = 6), mice treated with DSS (*n* = 10), DSS-treated mice + 0.05% αT supplement (*n* = 10) and DSS-treated mice + 0.05% γTmT supplement (*n* = 10))	IL-6 ↓ LBP ↓ *Lachnospiraceae UCG006* ↑ *Roseburia* ↑	[58]
Colorectal cancer	Animal	6–7-week-old male Balb/c mice (all mice were injected with AOM and distributed randomly into 3 groups: AIN-93G control group, δTE supplemented group and δTE-13′-COOh group)	IL-1β ↓ GM-CSF↓ MCP-1 ↓ *Firmicutes*: *Bacteroidetes* ↓ *Roseburia* ↑ *Eubacterium coprostanoligenes* ↑ *Clostridiales vadinBB60*↓ *Streptococcaceae* ↑ *Lactococcus* ↑ *Parabacteroides goldsteinii CL02T12C30* ↑	[74]

↓ = decrease; ↑ = increase.

## 5. Conclusions

The current findings serve as evidence where vitamin E is a promising therapeutic approach in delaying the progression of age-related diseases. This research has demonstrated that vitamin E influences the gut microbiota composition and leads to an improvement in the diseases studied. Hence, this illustrates that it is vital to discover an intervention capable of modulating the gut microbiome to buy time for the progression of a disease. However, one of the limitations is the limited research conducted to understand how vitamin E affects the gut microbiota and its connection with age-related diseases. Next, although these animal studies have demonstrated the positive effects of vitamin E, there are still a few research gaps which have not yet been addressed. Firstly, all research that studies the effect of vitamin E on gut microbiome and the relationship with age-related diseases was performed mainly on animals, but very little was performed on humans. Clinical trials should take into consideration the safe dosage of vitamin E supplementation. This is to avoid any side effects or harm being brought to humans. The safe dosage of vitamin E varies among diseases. For instance, in diabetes patients, the safe dosage of vitamin E is between 400 and 700 mg/day where no adverse effect has been reported [81]. Meanwhile, a study discovered that 2000 IU of vitamin E, which is equivalent to 1340 mg, reduced the progression of cognitive decline in study participants with no adverse effect to their health [82]. Moreover, studies should also be performed by comparing the effects of different vitamin E forms to detect which vitamin E form is the best in performing its role. Next, the age-related diseases discussed in this review have some similarities in the progression of disease. This includes the reduction in beneficial bacteria and SCFA levels along with the increase in pro-inflammatory cytokine production. Vitamin E has shown its beneficial activities, which include antioxidant and anti-inflammatory, throughout each study carried out using vitamin E. Thus, future studies may focus on reducing pathogenic bacteria and pro-inflammatory cytokine levels that contribute to inflammation, together with increasing the SCFAs levels in humans, through the consumption of an optimum safe dosage of vitamin E. Furthermore, the mechanism behind the modulation of vitamin E on the gut microbiome and the subsequent events giving rise to a particular age-related disease are not well established. Hence, more research studying the underlying biological pathways and other interactions involved is required to understand this area of study more deeply.

## Data Availability

Not applicable.
